# Application of High-Frequency Oscillations on Scalp EEG in Infant Spasm: A Prospective Controlled Study

**DOI:** 10.3389/fnhum.2021.682011

**Published:** 2021-06-10

**Authors:** Lisi Yan, Lin Li, Jin Chen, Li Wang, Li Jiang, Yue Hu

**Affiliations:** ^1^Department of Neurology, Children’s Hospital of Chongqing Medical University, Chongqing, China; ^2^Ministry of Education Key Laboratory of Child Development and Disorders, Chongqing, China; ^3^National Clinical Research Center for Child Health and Disorders, Chongqing, China; ^4^China International Science and Technology Cooperation Base of Child Development and Critical Disorders, Chongqing, China; ^5^Chongqing Key Laboratory of Pediatrics, Chongqing, China

**Keywords:** high-frequency oscillation, infantile spasm, quantitative analysis, time–frequency analysis, scalp electroencephalography

## Abstract

**Objective:**

We quantitatively analyzed high-frequency oscillations (HFOs) using scalp electroencephalography (EEG) in patients with infantile spasms (IS).

**Methods:**

We enrolled 60 children with IS hospitalized from January 2019 to August 2020. Sixty healthy age-matched children comprised the control group. Time–frequency analysis was used to quantify γ, ripple, and fast ripple (FR) oscillation energy changes.

**Results:**

γ, ripple, and FR oscillations dominated in the temporal and frontal lobes. The average HFO energy of the sleep stage is lower than that of the wake stage in the same frequency bands in both the normal control (NC) and IS groups (*P* < 0.05). The average HFO energy of the IS group was significantly higher than that of the NC group in γ band during sleep stage (*P* < 0.01). The average HFO energy of S and Post-S stage were higher than that of sleep stage in γ band (*P* < 0.05). In the ripple band, the average HFO energy of Pre-S, S, and Post-S stage was higher than that of sleep stage (*P* < 0.05). Before treatment, there was no significant difference in BASED score between the effective and ineffective groups. The interaction of curative efficacy × frequency and the interaction of curative efficacy × state are statistically significant. The average HFO energy of the effective group was lower than that of the ineffective group in the sleep stage (*P* < 0.05). For the 16 children deemed “effective” in the IS group, the average HFO energy of three frequency bands was not significantly different before compared with after treatment.

**Significance:**

Scalp EEG can record HFOs. The energy of HFOs can distinguish physiological HFOs from pathological ones more accurately than frequency. On scalp EEG, γ oscillations can better detect susceptibility to epilepsy than ripple and FR oscillations. HFOs can trigger spasms. The analysis of average HFO energy can be used as a predictor of the effectiveness of epilepsy treatment.

## Introduction

Extensive high-frequency oscillations (HFOs) occur in the neural network. HFOs appear on electroencephalography (EEG) with a frequency of 40–500 Hz. HFOs include gamma (γ; 40∼80 Hz), ripple (80∼200 Hz), and fast ripple (FR; 200∼500 Hz) oscillations (Ferrari-Marinho et al., 2020). The physiological neural network (Kramer and Cash, 2012) realizes temporary synchronization by γ oscillation, synchronizes nodes in the distant and contralateral hemispheres, and facilitates rapid and complex nerve conduction. Epileptic seizures occur due to excessive neuronal excitation in an imbalanced neural network and affect other related networks paroxysmally or constantly, leading to dysfunction. The clinical applications of HFOs include preoperative evaluation of epilepsy surgery, evaluation of seizure severity, evaluation of the efficacy of various methods of epilepsy treatment, evaluation of the severity of the pathological injury, and detection of susceptibility to epilepsy and seizures. Owing to the limitations of low amplitude and spatial distribution, HFOs are recorded using intracranial electrodes with a sampling frequency of > 2,000 Hz. An artificial analysis is still the gold standard for HFO analysis (Frauscher et al., 2017). However, artificial analysis is time-consuming, and due to its inevitable subjectivity, HFOs are not routinely used in clinical practice.

Conventional EEG can only record 0.5–70 Hz of EEG activity (narrow-frequency EEG), which results from a large number of neurons (over 1 million) involved in firing and synchronization in a wide space. Because of high- and low-frequency filtering inside the amplifier, important low- or high-frequency electrical activity may be attenuated or distorted, which is important in the context of cortical signal processing. One study showed that HFOs can also be recorded using scalp EEG. Scalp EEG in children with infantile spasms (IS) can record fast activity with a frequency of between 80 and 100 Hz (Ferrari-Marinho et al., 2020). A previous study (Kobayashi et al., 2010) of 10 children with a continuous spike-and-wave pattern during sleep showed HFOs on scalp EEG, which appeared simultaneously with the spinous wave with an HFO frequency range of 97.7–140.6 Hz. Patients with focal epilepsy also demonstrate γ and ripple oscillations on scalp EEG (Andrade-Valenca et al., 2011). The HFO ratio of intracranial records in adults and children is close, but the scalp HFO rate is 100-times higher in children with epilepsy compared with adults (Frauscher et al., 2017).

We enrolled patients with IS and used time–frequency analysis to quantitatively analyze HFO energy changes in different frequency bands before and after treatment to confirm the reliability of scalp EEG to analyze HFOs. We also aimed to clarify whether HFOs recorded by scalp EEG reflect epilepsy severity and evaluate the efficacy of drugs to provide a theoretical basis for better application of HFOs in the clinic.

## Materials and Methods

### Research Objects

Children who conformed to the diagnostic criteria for IS (Pellock et al., 2010) and who were hospitalized at the Children’s Hospital of Chongqing Medical University from January 2019 to August 2020 were enrolled. Follow-up was conducted through outpatient visits and telephone interviews after discharge.

The inclusion criteria were as follows: (1) onset age < 12 months; (2) typical clinical symptoms, including sudden or transient spasm of the neck, trunk, and limbs, either symmetrically or asymmetrically; (3) hypsarrhythmia or atypical hypsarrhythmia on interictal EEG; (4) developmental delay or retrogression; (5) aged from 3 to 12 months.

The exclusion criteria were as follows: (1) suspected or proven neurometabolic disease or degenerative brain disease (Angappan et al., 2019); (2) proven severe disease (e.g., dysfunction of the liver, kidney, or heart); (3) lack of normative treatment for various reasons; (4) request from parents to withdraw their child from clinical observation.

The normal control (NC) group included children who attended the physical examination center of the Children’s Hospital from January 2019 to August 2020. The age range of patients was 3–12 months. Video EEG (VEEG) results were reportedly normal.

This study is a prospective controlled study. The study was approved by the Ethics Committee of the Children’s Hospital of Chongqing Medical University, and families of children provided written informed consent.

### Collection and Analysis of Scalp EEG Data

We adopted the International 10–20 system (Nihon-Kohden, Tokyo, Japan), and the frequency of sampling was 1,000 Hz. VEEG was performed for 4 h with electromyography of the deltoid and quadriceps femoris simultaneously. Sleep persisted for at least 60 min. EEG data analysis was completed independently by two well-trained neuroelectrophysiological professionals who reached a consensus on EEG and HFO interpretation. VEEG in the IS group was graded according to Burden of Amplitudes and Epileptiform Discharges (BASED) scoring criteria (Mytinger et al., 2015). The BASED score was completed according to longitudinal bipolar montage.

The IS group were examined during the interictal period, including during the (1) sleep stage (the sleep spindle appears) with epileptic discharge (SED) and a 5-min EEG segment with the epileptic discharge with sleep spindles and low-amplitude epidermal myoelectricity; (2) wake stage with epileptic discharge (WED) and a 5-min EEG segment with the epileptic discharge with low-amplitude epidermal myoelectricity in the wake stage with the eyes closed; (3) Stages of the epileptic attack were also assessed, including pre-spasm (Pre-S) for 2 s, spasm (S) for 2 s, and post-spasm (Post-S) for 2 s. The NC group was examined during the interictal period, including during sleep stage with no epileptic discharge (SNED) and wake stage with no epileptic discharge (WNED).

A high-frequency analysis was performed using the average montage as a reference. The spike-wave analysis had the following settings: screen display, 10 s/page; sensitivity, 15∼30 μV/mm; low-frequency filtering, 0.53 Hz; high-frequency filtering, 70 Hz. Then, the spike mark was hidden and the parameters for HFOs were adjusted as follows: chart speed, 1∼2 s/page; sensitivity, 3∼5 μV/mm; low-frequency filtering, 80 Hz; high-frequency filtering, 300 Hz. An HFO was defined as one event containing at least four consecutive and regular oscillations with a higher frequency and amplitude compared with its surrounding background (Zijlmans et al., 2012). In one EEG recording, HFOs should appear at least twice in 5 min to ensure that similar oscillatory waves can be marked in 60 s of EEG and that the marking rate of false HFOs can be reduced.

Five-min EEG signals selected at the above time points were quantitatively analyzed for HFO signals. Decomposition was performed using MATLAB wavelet analysis, and γ (40∼80 Hz), ripple (80∼200 Hz), and FR (200∼300 Hz) oscillations were examined to perform the average energy analysis. The Morlet wavelet algorithm is described in previous studies (Xiang et al., 2014, 2015).

### Curative Evaluation

A short-term curative effect judgment was made after 4 weeks of treatment (Cao et al., 2011; Han et al., 2016), including clinical and EEG judgment. Clinical assessment classifications included “controlled” (seizure-free), “improved” (>50% reduction in the frequency of seizures), and “ineffective” (< 50% or no reduction in the frequency of seizures). The controlled and improved classifications were categorized as “effective.” The criteria for EEG improvement (Mytinger et al., 2015) were assessed by comparing EEG results pre-treatment versus 4 weeks after treatment. A BASED score of ≤3 was considered effective, while a BASED score of ≥ 4 was considered ineffective. Efficiency was calculated as follows: efficiency = (control + improved) ÷ total number of cases × 100%.

### Statistical Analysis

Statistical analysis was performed using SPSS 26.0 software. The Shapiro–Wilk method was adopted to test the normality of measurement data. Mean ± standard deviation is used to present data with a normal distribution, a paired *t*-test was used for intra-group comparison. Measurement data with a skewed distribution are expressed as median and interquartile range. Aligned Rank Transform (ART) is used to implement non-parametric liner mixed model in R (version 4.0.0) Package “ARToo” (version 0.11.0). The ARTool relies on a preprocessing step that “aligns” data before applying averaged ranks, after which point common ANOVA procedures and *post hoc* can be used (Wobbrock et al., 2011). Using Holm method to correct P value after comparison in ARTool (Elkin et al., 2021). Counting data is expressed as frequency. A P value of ≤ 0.05 was considered statistically significant.

## Results

### General Information

Sixty children with IS met the inclusion criteria (89 children were excluded), including 33 males and 27 females with a ratio of 1.22:1. The age range of patients was 3–12 months (mean age, 6.92 ± 2.39 months) (Table 1). The NC group consisted of 60 healthy age-matched children (32 males and 28 females with a ratio of 1.14:1). The age range of patients in the NC group was 3–12 months (mean age, 6.28 ± 2.38 months).

**TABLE 1:** Clinical features of the IS group (60 cases).

Project		Case
**Sex**		
	Male	33
	Female	27
**Perinatal risk factors**		
	Premature	4
	Asphyxia	8
	Hypoglycemia	1
	Pathological jaundice	1
**Onset to start of treatment**		
	< 1 month	30
	≥ 1 month	30
**Mode of seizure**		
	Cluster of spasms	57
	Partial, isolated spasm	3
**Head MRI**		
	Cerebromalacia	6
	Ventricular dilatation	4
	Delayed myelination	6
	Callosal agenesis	4
	Tuberosa sclerosis	7
	Subarachnoid hemorrhage	1
	Pachygyria	1
**VEEG**		
Pre-treatment	5 (BASED score)	51
	4 (BASED score)	9
Post-treatment	≥ 4 (BASED score)	14
	≤ 3 (BASED score)	16
**Gene**		
	14q11q12 novel copy number variation	1
	TSC2 *de novo* mutation	4
	STXBP1 *de novo* mutation	1
	SCN2A novel mutation	1
	Negative	7
**Medication**		
	Glucocorticoid	55
	Topiramate	44
	Valproic acid	29
	Two drugs combined	29
	More than two drugs combined	29
	Ketogenic diet	2

### Average Energy Analysis of HFOs on Scalp EEG in the IS Group Before Treatment

The HFO lead with the largest average energy is referred to as the responsible lead. Sixty children in the NC group demonstrated 204 responsible leads, with most being in the frontal lobe (79/204) and the temporal lobe (83/204). Sixty children with IS demonstrated 260 responsible leads, with most being in the temporal lobe (119/260) and the frontal lobe (94/260).

**FIGURE 1:**
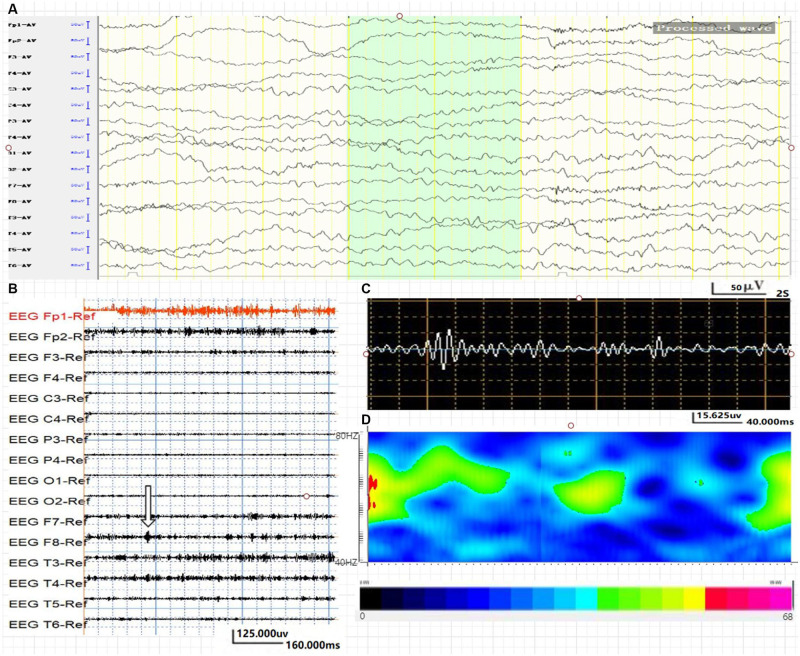
Average energy and time characteristics of γ oscillations in WNED (NC group). The waveform and energy diagram shows the energy and time characteristics of γ oscillations (40∼80 Hz) in WNED (NC group). **(A)** Scalp EEG of WNED (NC group). **(B,C)** 40∼80-Hz bandpass filter. **(C)** F8 lead signals. **(D)** Spectrum diagram reflects the cumulative time–frequency of the corresponding waveform.

**FIGURE 2:**
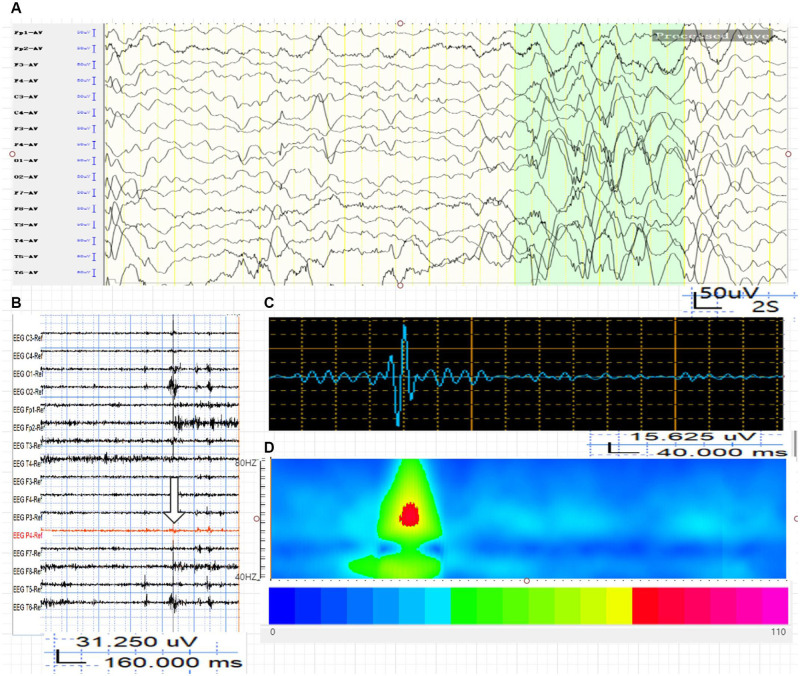
Average energy and time characteristics of γ oscillations in WED (IS group). The waveform and energy diagram shows the energy and time characteristics of γ oscillations (40∼80 Hz) in the WED (IS group). **(A)** Scalp EEG of WED (IS group). **(B,C)** 40∼80-Hz bandpass filter. **(C)** P4 lead signals. **(D)** The spectrum diagram reflects the cumulative time-frequency of the corresponding waveform.

**FIGURE 3:**
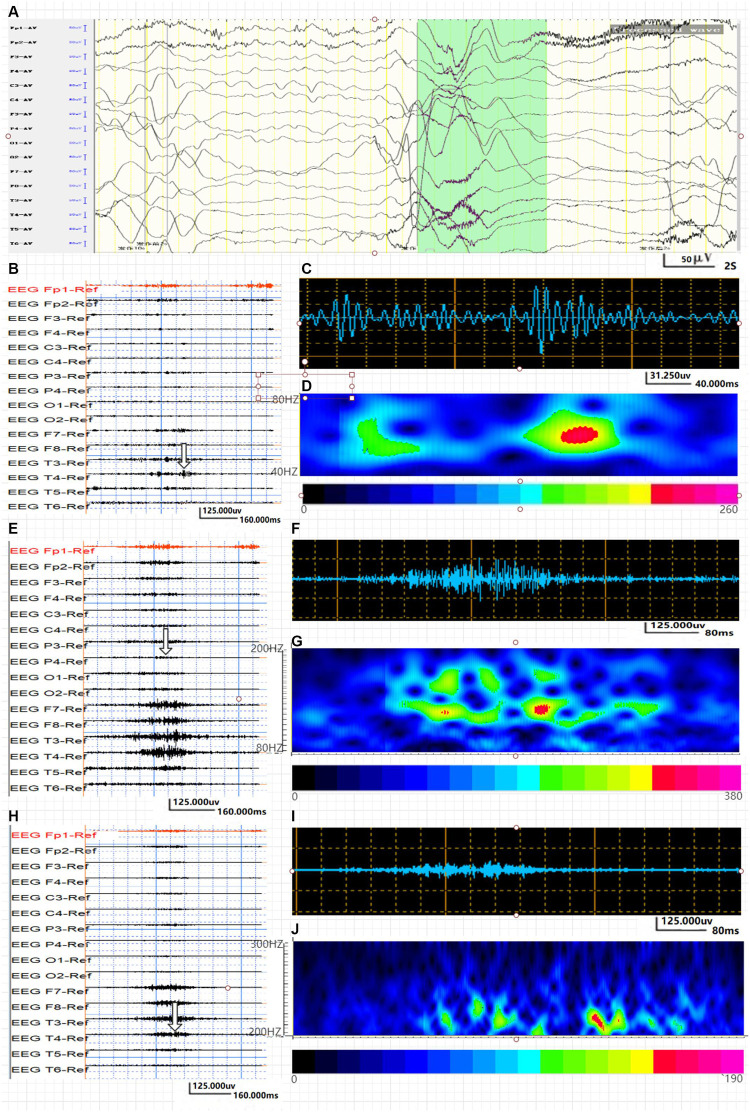
Average energy and time characteristics of γ, ripple, and fast ripple oscillations in the IS group. The waveform and energy diagram shows the energy and time characteristics of γ, ripple, and fast ripple oscillations. **(A)** Ictal EEG of the scalp in the IS group. **(B**,**C)** 40∼80-Hz bandpass filter. **(C)** γ oscillation in the O1 lead. **(D)** The spectrum diagram reflects the cumulative time–frequency of γ oscillations of the corresponding waveform. **(E,F)** 80∼200-Hz bandpass filter. **(G)** The spectrum diagram reflects the cumulative time-frequency of ripple oscillations of the corresponding waveform. **(H**,**I)** 200∼300-Hz bandpass filter. **(J)** The spectrum diagram reflects the cumulative time-frequency of fast ripple oscillations of the corresponding waveform. The characteristic feature of ripple and fast ripple oscillations is rhythmic burst, which is different from system artifacts, such as power noise and harmonics.

We compared the IS group with the NC group and found that the interaction effects of group (IS/NC group) × state (sleep/wake stage) × frequency (γ/ripple/FS) are statistically significant (*F*_2,590_ = 3.13, *P* = 0.045). After further pairwise comparison, it was found that in γ and sleep stage, the average energy of the IS group was significantly higher than that of the NC group (*P* < 0.01) (Figures 1–3 and Table 2).

**TABLE 2:** Comparison of the average HFO energy between/within the IS group and the NC group before treatment.

	γ	Ripple	FR
**NC group**			
Wake stage	73.99(50.16, 110.75)	37.09(23.81, 63.99)	12.44(9.41, 18.84)
Sleep stage	31.05(22.57, 51.09)^▲^	10.65(8.90, 14.57)^▲^	9.02(4.53, 9.76)^▲^
**IS group**			
Wake stage	89.87(62.67, 215.44)	40.47(26.06, 76.39)	18.59(9.48, 28.12)
Sleep stage	44.63(30.99, 170.19)^△▲^	12.91(10.45, 21.10)^▲^	9.30(4.42, 21.62)^▲^
Pre-S stage	92.09(45.78, 151.86)	34.28(19.30, 47.13)^★^	14.47(8.05, 41.56)
S stage	109.99(79.31, 144.83)^★^	42.26(21.45, 53.38)^★^	15.78(7.68, 29.69)
Post-S stage	103.17(59.50, 166.93)^★^	46.36(21.41, 86.68)^★^	23.36(8.66, 40.68)^★^

*△, Compared with the average HFO energy of sleep stage (NC group) in the same frequency band P < 0.01. ▲, Compared with the average HFO energy of wake stage (IS group) in the same frequency band P < 0.05. ★, Compared with the average HFO energy of sleep stage (IS group) in the same frequency band P < 0.05. P, Adjusted by Holm method.*

In the IS group, the interaction effect of state × frequency is statistically significant (*F*_8,462_ = 2.16, *P* = 0.030). After further pairwise comparison, it is found that the average energy of the sleep stage is lower than that of the wake stage in the same frequency bands (*P* < 0.05). In the γ band, the average energy of S and Post-S stage were higher than that of sleep stage (*P* < 0.05). In the ripple band, the average energy of Pre-S, S, and Post-S stage was higher than that of sleep stage (*P* < 0.05). In the FR band, only the average energy of the Post-S stage is higher than that of sleep stage (*P* < 0.05). In all frequency bands, there was no significant statistical difference between the average energy of the Pre-S/S/Post-S stage and the wake stage (*P* > 0.05) (Table 2).

In the NC group, the interaction effect of state × frequency was statistically significant (*F*_2,295_ = 41.22, *P* < 0.001). After further pairwise comparison, it is found that the average energy of the sleep stage is lower than that of the wake stage in the same frequency bands (*P* < 0.05) (Table 2).

### Average Energy Analysis of HFOs on Scalp EEG in Different Frequency Bands Between the Effective and Ineffective Groups Before and After Treatment

**FIGURE 4:**
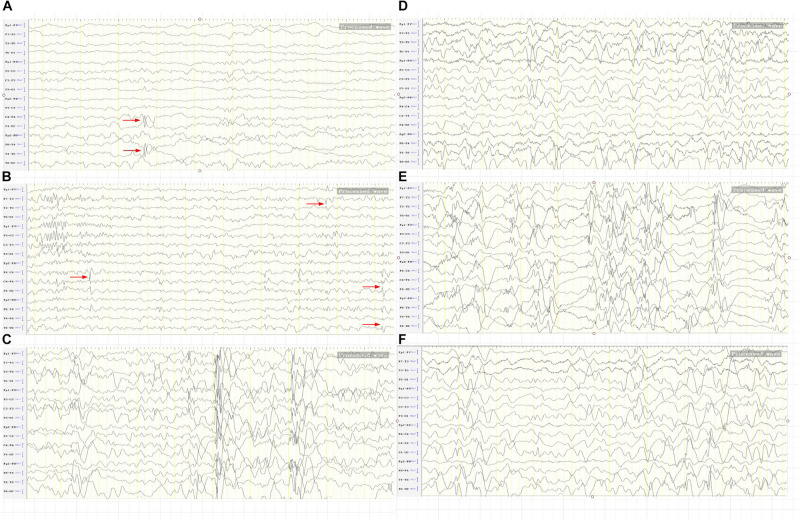
The BASED score in the IS group. **(A)** Two points. After treatment, spikes on the right posterior temporal region. **(B)** Three points. After treatment, spikes on the right central-parietal-posterior temporal region left middle temporal region and a slow wave amplitude of < 200 μV on the background. **(C)** Four points. After treatment, an epilepsy discharge of < 50% within 1 s of the most severe 5 min of EEG, and a slow wave amplitude of > 200 μV on the background. **(D)** Four points. After treatment, an epilepsy discharge of < 50% within 1 s of the most severe 5 min of EEG, and a slow wave amplitude of > 200 μV on the occiput. **(E)** Five points. Before treatment, an epilepsy discharge of > 50% within 1 s of the most severe 5 min of EEG. **(F)** Five points. Before treatment, a low wave amplitude of > 300 μV in the bilateral parietal posterior temporal region.

After normative treatment, 30 children (17 males) underwent VEEG after 4 weeks of treatment and were divided into an effective group (16 cases) and an ineffective group (14 cases) according to clinical efficacy and EEG score (Figure 4). The remaining 30 cases failed to complete follow-up. We statistically analyzed the average HFO energy during the interictal period in the ineffective and ineffective groups before treatment.

Before treatment, there was no significant difference in BASED score between the effective and ineffective groups [median(Q1, Q3), 5.00(5.00, 5.00) vs. 5.00(5.00, 5.00), respectively]. The interaction of curative efficacy (effective/ineffective) × state (sleep/wake stage) × frequency (γ/ripple/FS) is not statistically significant (*F*_2,140_ = 0.76, *P* = 0.469). However, the interaction of curative efficacy × frequency (*F*_2,140_ = 5.38, *P* = 0.005) and the interaction of curative efficacy × state (*F*_1,140_ = 5.95, *P* = 0.016) are statistically significant. After further pairwise comparison, it was found that the average energy of the effective group was lower than that of the ineffective group in the sleep stage (*P* < 0.05, Table 3).

**TABLE 3:** Comparison of the average HFO energy between the effective and ineffective group before treatment.

	Ineffective group	Effective group	*P*
**Stage**			
Wake stage	36.62(19.55, 74.59)	51.09(21.17, 85.35)	0.486
Sleep stage	18.98(11.21, 33.55)	13.22(8.11, 38.75)	0.041
**Frequency**			
γ	71.93(33.90, 206.56)	82.95(40.66, 110.17)	> 0.999
Ripple	21.60(12.99, 37.13)	23.74(12.41, 50.66)	> 0.999
FR	11.43(9.43, 25.60)	11.54(5.14, 18.11)	>0.999

*P, Adjusted by Holm method.*

We further analyzed the average HFO energy before and after treatment in 16 effective children in the IS group. The results show that the BASED score before treatment (mean, 4.75 ± 0.58) was higher compared with after treatment (mean, 1.31 ± 1.25) (*P* < 0.01). The interaction between time (before/after treatment) × state (sleep/wake stage) × frequency (γ/ripple/FS) is not statistically significant (*F*_2,162_ = 0.84, *P* = 0.435). The interactions of time × frequency (*F*_2,162_ = 1.96, *P* = 0.145) and time × state (*F*_2,162_ = 2.34, *P* = 0.128) were also not statistically significant. The main effect before and after treatment was also not statistically significant *(F*_1,162_ = 0.03, *P* = 0.871).

## Discussion

HFOs are weaker than normal-frequency EEG signals, with lower amplitude and a shorter duration. Their characteristics cannot be accurately expressed by traditional time–domain or frequency–domain analyses (Modur, 2014; Frost et al., 2015; Zijlmans et al., 2017). The wavelet transforms in the time–frequency analysis is applied in signal analysis and processing (e.g., signal singularity detection, time-varied filtering, and pattern recognition) (Yong and Shengxun, 1996). The Morlet wavelet algorithm features good time resolution and based on Gaussian function, it allows signals to have phase fluctuations within a certain range. Using this approach, the instantaneous spectral characteristics of EEG signals with non-stationary characteristics are observed, and the quantitative analysis of HFOs in epileptic brain tissue can be realized (Xiang et al., 2015). Average energy reflects the functional brain state throughout the time window, while maximum energy reflects the burst of maximum energy at a specified time point in the time window; however, maximum energy reflects different functional brain states with poor variation stability and recognizes functional changes with lower sensitivity than average energy. Therefore, average energy is used to analyze the functional brain state and to assess seizures. Non-invasive scalp EEG is disturbed by electromyographic signals, which are a well-known source of high-frequency activity, with the major signal frequency being concentrated at 20–150 Hz. We were careful to eliminate artifacts in the present data. To eliminate artifacts, EEG data analysis was completed independently by two experienced neuroelectrophysiologists who reached a consensus on EEG and HFO interpretation. Low-amplitude surface electromyographic signal segments were selected, and manual and automatic detection were performed simultaneously. The reliability and accuracy of the analysis methods were confirmed (Leiken et al., 2014). The spectrum diagram reflects the accumulated time–frequency of the HFO of the corresponding waveform, and HFO is a rhythmic outbreak that is different from system artifacts, such as power supply noise and its harmonics.

The skull does not filter high-frequency signals, but its thickness and resistance attenuate the conduction of intracranial EEG signals (Gotman, 2010). Therefore, it is generally believed that high-frequency signals are difficult to record using scalp EEG. At lower noise levels, ripples can be recorded on scalp EEG, but the amplitude is 10-times lower than with intracranial EEG (von Ellenrieder et al., 2014). Only a few studies show that scalp EEG can record FRs (Mooij et al., 2017). In this study, the average energy of HFOs confirmed that scalp EEG in patients with epilepsy and healthy children could record EEG signals in three frequency bands (γ, ripple, and FR), which mainly appear in the temporal and frontal lobes. HFOs are affected by sleep. Studies show that the HFO rate is highest during non-rapid eye movement (NREM) sleep and lowest during rapid eye movement (REM) sleep and awake stages. The area of HFOs in NREM sleep is larger (von Ellenrieder et al., 2017). Conversely, in the present study, the average HFO energy of the sleep stage is lower than that of the wake stage in the same frequency bands in both the NC and IS groups (*P* < 0.05). Thus, physiological and pathological HFOs have similar sleep balance characteristics. Besides, disturbance of scalp EEG by motor artifacts or myoelectrical activity during the waking period cannot be completely excluded.

HFOs reflect epilepsy severity. Boran et al. studied HFOs using scalp EEG before and after surgery in 11 children with intractable epilepsy undergoing surgery. The incidence of scalp HFOs positively correlated with seizure frequency and decreased after epilepsy surgery (Boran et al., 2019). In patients with atypical benign partial epilepsy, ripples recorded on scalp EEG were related to increased seizure frequency in negative myoclonus and atypical absence seizure (Qian et al., 2016). Nicole et al. discovered that the number of ripples on Rolandic spikes positively correlated with seizure frequency, suggesting that ripples on Rolandic spikes reflect seizure severity (van Klink et al., 2016). In epileptic spasms, whether clinical spasms occur is strongly linked to the amplitude of HFOs in the Rolandic area (Qian et al., 2016). In this study, it was found that in γ and sleep stage, the average HFO energy of the IS group was significantly higher than that of the NC group (*P* < 0.01). Previous intracranial electrode research in animals proved that ripple and FR oscillations can appear in the normal or epileptic hippocampus simultaneously, and the main difference between the two states is in HFO energy. Therefore, HFOs energy can more accurately distinguish physiological from pathological HFOs than frequency (Song et al., 2016). It may be considered that the origin of ripple and FR is relatively small, coupled with the distance and resistance of the skull, further leading to the attenuation of this weak signal. On scalp EEG, γ oscillations can better detect susceptibility to epilepsy than ripples and FRs.

Pathological HFOs reflect neuronal firing, which is the basis for convulsions (Lévesque et al., 2011; Zijlmans et al., 2011). From the Pre-S to the S period, the oscillation frequency gradually increases over time. Changes in ripple and FR energy coincide with epileptic seizure stages, which have an indicative effect on seizures (Song et al., 2016). Pearce et al. (2013) discovered that HFOs begin to change 30 min before a seizure. Moreover, HFOs appear and increase significantly before spasms, suggesting that HFOs can trigger spasms (Nariai et al., 2011). The present study suggests that in the γ band, the average HFO energy of S and Post-S stage were higher than that of sleep stage (*P* < 0.05). In the ripple band, the average HFO energy of Pre-S, S, and Post-S stage was higher than that of sleep stage (*P* < 0.05).

Typical EEG traces of patients with IS manifest as hypsarrhythmia. The BASED score is a simplified EEG scoring standard based on the weight of amplitude and epileptiform discharges. A score is produced by analyzing the most severe 5 min of EEG signals in children with IS (0 and 1 point not applicable). According to the BASED score, the standard for judging hypsarrhythmia is a BASED score of 4 or 5, and no hypsarrhythmia is concluded with a score of ≤ 3. Compared with traditional EEG, the BASED score boasts better consistency among scorers (Mytinger et al., 2015); thus, a judgment of hypsarrhythmia in patients with IS is more accurate and reliable than with traditional EEG. In this study, there was no significant difference in BASED score between the effective and ineffective groups before treatment. We further analyzed the average HFO energy before and after treatment in 16 effective children in the IS group. The results show that the BASED score before treatment (mean, 4.75 ± 0.58) was higher compared with after treatment (mean, 1.31 ± 1.25) (*P* < 0.01).

Boran et al. found that the frequency of HFOs is positively correlated with the frequency of epileptic discharge (Boran et al., 2019). Patients with active partial epilepsy have a higher frequency of HFOs (accuracy rate, 88%). HFOs have clear monitoring significance for the response to drug treatment in patients with epileptic encephalopathies, such as epileptic encephalopathy with continuous spike-and-wave during sleep and variants of benign childhood epilepsy with centrotemporal spikes. The prognosis of patients with epilepsy undergoing methylprednisolone therapy is closely related to the number of HFOs on scalp EEG (Qian et al., 2016; Gong et al., 2018). Dezhi et al. studied HFOs in 22 children with persistent spike-wave encephalopathy during sleep who received methylprednisolone treatment. HFOs disappeared from scalp EEG in children treated with hormones, but children with no response or recurrence after hormone therapy showed persistent HFOs (Cao et al., 2019). In the study, the interaction of curative efficacy × frequency and the interaction of curative efficacy × state are statistically significant. The average HFO energy of the effective group was lower than that of the ineffective group in the sleep stage (*P* < 0.05). Therefore, the evidence supports the analysis of average HFO energy can be used as a predictor of the effectiveness of epilepsy treatment. For the 16 children deemed “effective” in the IS group, the average HFO energy of three frequency bands was not significantly different before compared with after treatment. IS is a type of developmental epileptic encephalopathy. Even if seizures are controlled and EEG observations improve, varying degrees of cognitive dysfunction may still be present (Specchio and Curatolo, 2021). HFOs are involved in memory formation and information processing (Alkawadri et al., 2014). Therefore, whether the high-energy HFOs of IS patients after treatment is related to cognitive dysfunction needs to be further confirmed.

The advantages of measuring scalp HFOs include non-invasiveness and convenience. However, HFO signals are weak, and interference and artifacts are common. Some scholars have questioned the feasibility of scalp HFO recordings (Thomschewski et al., 2019). Research on the simultaneous recording of EEG signals using scalp and intracranial electrodes has confirmed that scalp HFO signals originate from the cortex. At present, scalp HFOs cannot be directly applied to guide the diagnosis and treatment of epilepsy, and their reliability and stability cannot be completely determined. However, as a non-invasive examination, this approach is highly convenient. Both animal and clinical trials have confirmed that the HFOs automatic analysis and detection system we used is fast and accurate (Xiang et al., 2014; Song et al., 2016; von Ellenrieder et al., 2017). Although the analysis results cannot completely exclude the interference of myoelectrical activity, they can provide a reference for clinical exploration of non-invasive detection of HFOs.

## Data Availability Statement

The raw data supporting the conclusions of this article will be made available by the authors, without undue reservation.

## Ethics Statement

The studies involving human participants were reviewed and approved by Ethics Committee of the Children’s Hospital of Chongqing Medical University. Written informed consent to participate in this study was provided by the participants’ legal guardian/next of kin.

## Author Contributions

LY and LL were responsible for the material preparation. JC, LW, and LY collected and analyzed the data. LY and YH wrote the first draft of the manuscript. LJ reviewed the manuscript. All authors contributed to the study’s conception and design, commented on previous versions of the manuscript, and read and approved the final manuscript.

## Conflict of Interest

The authors declare that the research was conducted in the absence of any commercial or financial relationships that could be construed as a potential conflict of interest.
